# Increased expression of chymase in inflammatory polyps in elderly patients with functional bowel disorder

**DOI:** 10.3892/etm.2013.1444

**Published:** 2013-12-11

**Authors:** JIAN-MING RONG, HONG-ZAN JI, XIAO-WEI WU, QUAN SUN, MEI-XIA GUO, XIAO-BING XU, FANG-YU WANG

**Affiliations:** 1 Department of Geratology, No.454 Hospital of PLA, Nanjing, Jiangsu 210002, P.R. China; 2 Department of Gastroenterology, Nanjing Jin Ling Hospital, Nanjing, Jiangsu 210002, P.R. China

**Keywords:** chymase, functional bowel disorder, elderly, intestinal inflammatory polyps

## Abstract

Chymase, a chymotrypsin-like protease, is a non-angiotensin-converting enzyme (ACE) angiotensin II (Ang II)-generating enzyme. The aim of the present study was to investigate whether chymase activity was increased in inflammatory polyps of elderly patients with functional bowel disorder (FBD). This study enrolled 45 elderly patients with FBD and 44 healthy control individuals. Expression of chymase in intestinal mucosa was assessed using fluorescence quantitative polymerase chain reaction and immunohistochemistry (IHC). IHC showed an increased number of chymase-positive mast cells in inflammatory polyps than in healthy intestinal mucosa (P<0.05). Compared with healthy mucosa, expression of chymase at the mRNA and protein level was significantly higher in inflammatory polyps. The frequencies of the chymase GG genotype and the G allele type were higher in the intestinal mucosa of patients with FBD compared with healthy controls (66.67 versus 40.91%, 81.11 versus 63.63%, both P<0.05). The frequency of the G allele type in the intestinal mucosa of the C4 subgroup of FBD was higher than that in the control group. However, in other FBD subgroups, there was no difference between patients and controls. Based on the fact that enhanced chymase expression was observed in inflammatory polyps of elderly patients with FBD relative to those in healthy controls, it was concluded that chymase has a significant role in the pathogenesis of inflammatory polyps in elderly patients with FBD.

## Introduction

Functional bowel disorder (FBD) is the generic term for disorders of bowel motor and secretory function in the absence of organic changes. These disorders are diagnosed according to symptoms following the exclusion of lesions caused by inflammation, infection, tumor and other structural disorders (1,2). The incidence of FBD in the elderly is ~53%, although in patients >75 years, it is even higher (3). Inflammatory bowel polyps are benign proliferative lesions characterized by increased stromal cells and infiltration of inflammatory cells (4,5). Chymase, a chymotrypsin-like protease, is a non-angiotensin-converting enzyme (ACE) angiotensin II (Ang II)-generating enzyme with low expression in healthy intestinal mucosa (6,7). The expression of chymase has been shown to be upregulated in response to inflammatory stimulation, indicating its possible role in the pathogenesis of intestinal inflammatory polyps (8,9). In the present study, chymase expression was evaluated in intestinal inflammatory polyps and its correlation with the pathogenesis of intestinal inflammatory polyps in elderly patients with FBD was analyzed.

## Materials and methods

### Patients

Between August 2005 and August 2008, 45 consecutive outpatients of the Nanjing Jin Ling Hospital (Nanjing, China) were enrolled in this study. The study population comprised 28 males and 17 females (average age, 76.53±8.37 years) with inflammatory polyps and FBD. The study was conducted in accordance with the Declaration of Helsinki and approval was obtained from the Ethics Committee of Nanjing Jin Ling Hospital. Written informed consent was obtained from all participants. All cases were diagnosed according to the Rome III process (10) and divided into four subgroups. The characteristics of each subgroup are shown in Table I. A total of 44 healthy individuals (32 males, 12 females; average age, 76.68±6.41 years) were selected as controls.

All participants with demographic and clinical data signed the informed consent form. Ultrasound, computed tomography (CT) scan and colonoscopy were performed to exclude gastrointestinal organic diseases and other systemic diseases. None of the patients had received immunologic treatment in the three months prior to enrollment. Serial sections of paraffin-embedded intestinal mucosa tissues confirmed by pathologists were collected by biopsy during colonoscopy.

### Immunohistochemistry (IHC)

IHC staining was performed using a Streptavidin-Peroxidase (SP) kit (SP-9000, anti-mouse/anti-rabbit IgG; Zhongshan Laboratories, Zhongshan, China) (11). In brief, slides were dewaxed in xylene, rehydrated in alcohol, and incubated in 0.3% (v/v) hydrogen peroxide in methanol to block endogenous peroxidase activity. Antigens were retrieved by microwaving the sample on high-power for 15 min with two 5-min intervals using a 0.01-M sodium citrate buffer. Subsequent to incubating with normal goat serum for 1 h, the sections were incubated with an anti-human chymase polyclonal antibody (dilution 1:50; Zhongshan Belling Biotechnology Co., Ltd., Zhongshan, China) overnight at 4°C. The sections were then incubated with biotin-labeled goat anti-rabbit serum (1:2,000; Zhongshan Dongqiang Laboratories Co. Ltd., Zhongshan, China) for 30 min. Subsequent to washing with phosphate-buffered saline (PBS), the sections were treated with diaminobenzidine (DAB). The sections were then counterstained with hematoxylin, rinsed, dehydrated, and mounted. Sections incubated with PBS instead of primary antibody were used as negative controls. All sections were examined and scored independently by two experienced pathologists who had no knowledge of clinical or pathological information regarding the samples.

### Quantitative polymerase chain reaction (PCR)

Total RNA was isolated from tissues with TRIzol reagent (Invitrogen Life Technologies, Carlsbad, CA, USA) according to the manufacturer’s instructions (11). The reverse transcription reaction was performed using the First-Strand cDNA Synthesis kit (MBI Fermentas, Vilnius, Lithuania) according to the manufacturer’s instructions. Chymase primer sequences were as follows: 5′-GGAAATGTGAGCCAGATAGTGCAGTC-3′ (forward); 5′-AATCCGGAGCTGGAGAACTCTTGTC-3′ (reverse). The TaqMan^®^ stem-loop quantitative PCR method was used to assess chymase expression with kits from Applied Biosystems (Foster City, CA, USA). All quantitative PCR experiments were performed on a Chromo4™ Real-Time PCR Detection System (Bio-Rad, Hercules, CA, USA). PCR conditions were as follows: 93°C for 2 min, 93°C for 45 sec, 63°C for 45 sec, 35 cycles. CT values were applied to determine the mRNA level of target genes in each sample.

### Statistical analysis

Numerical count data are presented as the mean ± standard deviation. P<0.05 was considered to indicate a statistically significant difference. All statistical tests, including the Student’s t-test, χ^2^ test and Fisher’s exact test, were two-sided and performed with SAS statistical software (version 9.1; SAS Institute, Cary, NC, USA).

## Results

### IHC

**Figure 1:**
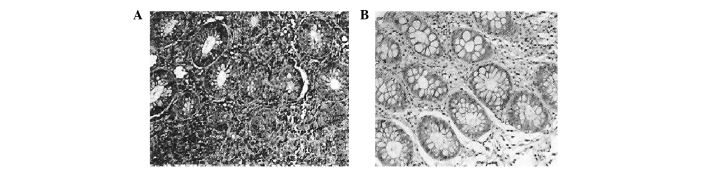
Chymase expression in (A) inflammatory polyps and (B) normal tissue with hematoxylin and eosin staining. Magnification, ×200.

Chymase expression was evaluated by IHC in 45 patients with inflammatory polyps and FBD compared with healthy controls. According to the present study, chymase was expressed predominantly in the cytoplasm of mucosa cells (Fig. 1). In addition, chymase-positive mast cells were distributed diffusely in the intestinal mucosa. An increased number of chymase-positive mast cells were observed in inflammatory polyps compared with healthy intestinal mucosa (P<0.05).

### Chymase mRNA level

To determine chymase mRNA expression, quantitative PCR was performed. As shown in Table II, 45 cases of inflammatory polyps had increased chymase mRNA levels compared with healthy controls. However, there were no significant differences among FBD subgroups.

### Chymase polymorphism analyses

The chymase genotype in patients with intestinal inflammatory polyps was further determined by PCR-restriction fragment length polymorphism (RFLP). It was shown that the frequency of the GG genotype in the intestinal mucosa of patients with FBD was significantly higher than that in healthy controls (66.67 versus 40.91%, P<0.05). A similar tendency was observed in the G allele type (81.11 versus 63.63%, P<0.05). The frequency of the AA genotype in patients with FBD was significantly lower than that in healthy controls (4.44 versus 13.6%, P<0.05). There were no significant differences in the frequency of the AG genotype and the A allele type between patients and controls (28.89 versus 45.45%, 18.89 versus 36.36%, both P>0.05).

The frequencies of different chymase genotypes in subtypes of FBD were further analyzed. It was observed that the frequency of the G allele type in the intestinal mucosa of the C4 subgroup was significantly higher than that in controls. However, in other subgroups, there was no difference between patients and controls (Table III).

**Figure 2:**
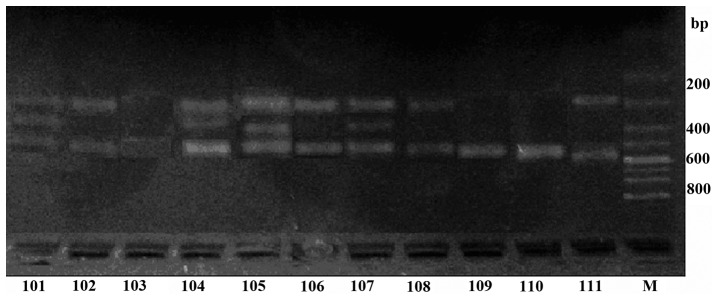
Electrophoresis results for samples. Lanes 101–103, healthy controls; lanes 104–111, patients with functional bowel disorder; M, marker.

The results of the electrophoresis are shown in Fig. 2. Samples in lanes 101–103 were from healthy controls, while samples in lanes 104–11 were from patients with FBD. The genotype of lanes 101, 104, 105 and 107 was AG heterozygote, while lanes 109 and 110 were AA homozygote and lanes 102, 103, 106, 108 and 111 were GG homozygote. The density of the lanes from patients with FBD was higher than the control lanes. The chymase G allele was observed as two bands at 467 and 186 bp, the AA homozygous genotype as one band at 654 bp, and the AG heterozygous genotype as three bands at 654, 467 and 186 bp (Fig. 2).

## Discussion

FBD is common in the elderly and often seriously affects quality of life. The molecular mechanism of neuroendocrine system abnormalities in FBD has become a particular focus of study (12). The effects of brain-gut peptides on the intestinal tract partly account for the pathology of FBD.

Chymase is a non-ACE that functions in the conversion of Ang I to Ang II in the non-circulating ACE pathway. Chymase is frequently present in secretion granules of mast cells, which are widely distributed in the intestinal mucosa, blood vessels, heart and other tissues (13). In our previous study, elevated chymase levels were observed in patients with FBD (14). Intestinal polyps are usually detected in FBD. In the present study, IHC staining was performed to determine the expression of chymase in mast cells of inflammatory bowel polyps and healthy mucosa. It was observed that, in inflammatory polyps, there was a significantly greater number of chymase-positive mast cells than in controls. These results suggested a close correlation between chymase levels and proliferation of inflammatory polyps.

In recent years, the correlation between mast and interstitial cells has raised particular concern. Chymase-producing mast cells are the precursors of interstitial cells. Therefore, with the proliferation of intestinal mucosa, the number of mast cells increases. In response to inflammatory stimuli, mast cells degranulate and chymase accumulates in intestinal tissues. The increased chymase converts Ang I into Ang II, and Ang II induces functional disorder of smooth muscle in the intestinal tract, which leads to the exacerbation of FBD (15,16).

The present study showed that chymase was overexpressed in inflammatory polyps at the mRNA and protein level. In an earlier study, we demonstrated that elevated chymase levels associated with hypertension induced target organ damage, including myocardial hypertrophy, modest elevated creatinine levels and microalbuminuria (3). Accumulation of chymase may also induce microvascular lesions in the intestinal tract, which may stimulate neuroendocrine cells to degranulate and cause functional disorder.

Currently available literature indicates that RAS dysfunction in abdominal sympathetic ganglia and the central nervous system is important in the pathogenesis of FBD (17). RAS overexpression in non-circulating tissues and its hyperactivity may stimulate angiogenesis, thus increasing the proliferation of polyps, and this may induce gene mutation and the development of malignancy (18).

Chymase has been demonstrated to be a potential target in the blockade of organ damage. Chymase inhibitors have shown efficacy in the intervention of aortic aneurysm, diabetic retinopathy, cardiac dysfunction and fibrosis. In gastrointestinal diseases, administration of dextran sulfate sodium (DSS) to mice yielded a significant increase in chymase activity (19–22). Thus, data from the present and previous studies suggest that chymase is involved in intestinal inflammatory diseases and that it may be a potential therapeutic target for patients with FBD.

## Figures and Tables

**Table I:** Characteristics of patients with FBD.

Category	Cases (n)	Age (years)	History (years)
Irritable bowel syndrome (C1)	8 (M 5, F 3)	76.53±8.37 (65–87)	9.05±6.99 (2–26)
Functional (C2)	10 (M 6, F4)	77.23±7.84 (65–87)	9.37±8.23 (5–36)
Functional constipation (C3)	14 (M 9, F 5)	77.37±6.18 (65–88)	10.44±6.25 (4–36)
Functional diarrhea (C4)	13 (M 8, F 5)	77.17±6.18 (65–87)	9.99±8.17 (2–36)

FBD, functional bowel disorder; M, male; F, female.

**Table II:** Chymase mRNA level in each group.

Category	Cases (n)	Chymase mRNA level
Control	44	0.60±0.11
FBD	45	0.81±0.10^a^
C1 subgroup	8	0.82±0.11
C2 subgroup	10	0.84±0.10
C3 subgroup	14	0.80±0.09
C4 subgroup	13	0.80±0.11

a Compared with control P<0.05.

FBD, functional bowel disorder.

**Table III:** Frequencies of chymase genotypes.

		Genotype (%)			Allele (%)	
			
Category	Cases (n)	AA	AG	GG	A	G
Control	44	6 (13.63)	20 (45.45)	18 (40.91)	32 (36.36)	56 (63.63)
FBD	45	2 (4.44)^a^	13 (28.89)	30 (66.67)^a^	17 (18.89)	73 (81.11)^a^
C1 subgroup	8	1 (12.50)	1 (12.50)	6 (75.00)	3 (18.75)	13 (81.25)
C2 subgroup	10	1 (10.00)	3 (30.00)	6 (60.00)	5 (25.00)	15 (75.00)
C3 subgroup	14	0 (0.00)	5 (35.71)	9 (64.29)	5 (17.86)	23 (82.14)
C4 subgroup	13	0 (0.00)	4 (30.77)	9 (69.23)	4 (15.38)	22 (84.62)^a^

a Compared with control P<0.05.

FBD, functional bowel disorder.
